# Pharmacological Correction of Stress-Induced Gastric Ulceration by Novel Small-Molecule Agents with Antioxidant Profile

**DOI:** 10.1155/2014/217039

**Published:** 2014-02-09

**Authors:** Konstantin V. Kudryavtsev, Anna O. Markevich, Oleksandr V. Virchenko, Tetyana M. Falalyeyeva, Tetyana V. Beregova, Lyudmyla I. Ostapchenko, Dmitry V. Zabolotnev, Nikolay S. Zefirov

**Affiliations:** ^1^Department of Chemistry, M.V. Lomonosov Moscow State University, Moscow 119991, Russia; ^2^Institute of Physiologically Active Compounds, Russian Academy of Sciences, Chernogolovka 142432, Russia; ^3^Institute of Biology, Taras Shevchenko National University of Kyiv, Kyiv 03022, Ukraine

## Abstract

This study was designed to determine novel small-molecule agents influencing the pathogenesis of gastric lesions induced by stress. To achieve this goal, four novel organic compounds containing structural fragments with known antioxidant activity were synthesized, characterized by physicochemical methods, and evaluated *in vivo* at water immersion restraint conditions. The levels of lipid peroxidation products and activities of antioxidative system enzymes were measured in gastric mucosa and correlated with the observed gastroprotective activity of the active compounds. Prophylactic single-dose 1 mg/kg treatment with (2-hydroxyphenyl)thioacetyl derivatives of *L*-lysine and *L*-proline efficiently decreases up to 86% stress-induced stomach ulceration in rats. Discovered small-molecule antiulcer agents modulate activities of gastric mucosa tissue superoxide dismutase, catalase, and xanthine oxidase in concerted directions. Gastroprotective effect of (2-hydroxyphenyl)thioacetyl derivatives of *L*-lysine and *L*-proline at least partially depends on the correction of gastric mucosa oxidative balance.

## 1. Introduction

**Figure 1:**
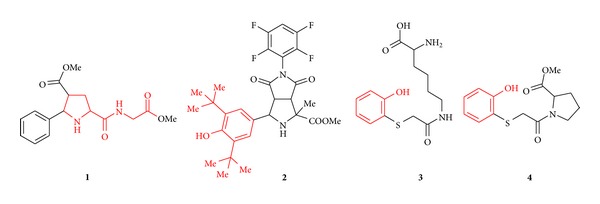
Structural formula of small-molecule agents synthesized and studied in the present work. Substructures with known antioxidant activity are shown in red.

The relationship between severe physiological stress and gastrointestinal (GI) ulceration is well recognized. Different types of GI injuries ranging from superficial to focal deep mucosal damages are considered as stress-related mucosal disease (SRMD) [1]. Stress ulcers differ from ordinary peptic ulcers in risk factors and symptoms. Usually SRMD is not associated with abdominal pain as in the case of peptic ulcers and induced lesions remit after the patient recovers. Common antiulcer medicines like histamine_2_-receptor antagonists (H_2_RAs) and proton pump inhibitors (PPI) have several drug interactions and adverse effects that limits their usage for the prevention of SRMD [1, 2]. There are strong suggestions that not only physical but also psychological stress is an important pathogenic factor for gastric ulceration [3]. Accordingly, development of novel agents influenced stress-related GI lesions is highly desired. Typically, primary mucosal erosions are referred to as stress-related injury (SRI) and, namely, stress ulcers represent focal deep mucosal damages with a high risk for bleeding. The main risk factors of SRMD bleedings are respiratory failure and coagulopathy [1]. Insufficient blood microcirculation in the upper GI tissues is considered as the major cause of mucosal defense reduction leading to the ulcer formation. Reactive oxygen species (ROS), such as superoxide anion O_2_^•−^, hydrogen peroxide H_2_O_2_, and hydroxyl radical HO^•^, accompany ischemic tissue events and are suggested as mediators of GI injuries of different etiology including stress-induced lesions [4]. ROS trigger lipid peroxidation (LPO) with subsequent loss of membrane fluidity, weakened ion transport and membrane integrity, and finally loss of cellular functions. Attenuating of ROS has been detected for several gastroprotective small-molecule drugs, such as rebamipide [5], melatonin [6], and omeprazole [7], and could be considered as an approach for the treatment of ulcerative GI pathologies. To determine correlations between oxidative and ulcerogenic factors, we studied effects of novel small-molecule organic compounds **1**–**4** (Figure 1) on SRI and ulceration progress in rats subjected to water immersion restraint as a stress conditions model.

## 2. Materials and Methods

### 2.1. Synthesis of Small-Molecule Compounds

Reagents were obtained from Alfa Aesar and used without further purification unless otherwise stated. Solvents were dried using standard procedures. Reactions were monitored by thin layer chromatography (TLC) on precoated silica gel plates (Sorbfil) with a UV indicator. Melting points were determined in open capillary and are uncorrected. ^1^H NMR and ^13^C NMR spectra were recorded with a Bruker Avance 400 MHz spectrometer. The chemical shifts (**δ**) are reported in parts per million upfield using residual signals of solvents as internal standards. Coupling constants (*J* values) were measured in hertz (Hz). Combustion analyses were performed with a Carlo Erba CHN analyzer.

#### 2.1.1. Methyl (2R*,3S*,5S*)-5-((2-Methoxy-2-oxoethyl)carbamoyl)-2-phenylpyrrolidine-3-carboxylate (**1**)

Under argon atmosphere 2.47 mL (1.793 g, 18 mmol) of NEt_3_ was added to a suspension of 2.900 g (16 mmol) of glycylglycine methyl ester hydrochloride and 3.500 g (29 mmol) of MgSO_4_ in 30 mL of CH_2_Cl_2_. After 1 h stirring at rt benzaldehyde (1.592 g, 15 mmol) was added and the resulted mixture was stirred at rt overnight. The solid was removed by filtration, the filtrate was concentrated, and the residue was redissolved in 30 mL of toluene and filtered again. Methyl acrylate (1.463 g, 17 mmol), AgOAc (2.660 g, 16 mmol), and 2.40 mL (1.742 g, 17 mmol) of NEt_3_ were added sequentially to the filtrate under argon atmosphere and stirring. The suspension was stirred for 24 h at rt. The mixture was diluted with 50 mL of CH_2_Cl_2_ and washed with saturated NH_4_Cl (2 × 10 mL) and H_2_O (2 × 10 mL). The organic phase was dried (Na_2_SO_4_), concentrated, and dissolved in methanol. The slow stream of HCl was passed through the methanolic solution under stirring at 0°C. White precipitate of the hydrochloride of the title product was filtered and dried. Yield 54% (2.890 g), white powder, mp 170–180°C. ^1^H NMR (400 MHz; DMSO-d_6_): **δ** 9.32 (t, *J* 5.6, 1H, N*H*CH_2_COOCH_3_), 7.39 (s, 5H, Ar), 5.09 (d, *J* 8.6, 1H, H-2), 4.52 (t, *J* 9.2, 1H, H-3), 4.08–3.96 (m, 2H, NHC*H*_*2*_COOCH_3_), 3.74–3.67 (m, 1H, H-5), 3.68 (s, 3H, OC*H*_*3*_), 3.28 (s, 3H, OC*H*_*3*_), 2.76 (ddd, *J* 13.5, 7.7, 7.6, 1H, H-4), 2.44–2.36 (m, 1H, H-4). Anal. Calcd. for C_16_H_21_N_2_O_5_Cl: C, 53.86; H, 5.93; N, 7.85. Found: C, 53.68; H, 5.79; N, 7.76.

#### 2.1.2. Methyl (1S*,3R*,3aS*,6aR*)-3-(3,5-Di-tert-butyl-4-hydroxyphenyl)-1-methyl-4,6-dioxo-5-(2,3,5,6-tetrafluorophenyl)octahydropyrrolo[3,4-c]pyrrole-1-carboxylate (**2**) Was Synthesized as Described in [8]

Yield 54%, white powder, mp 220–222°C. ^1^H NMR (400 MHz; CDCl_3_): **δ** 7.21–7.13 (m, 1H, Ar), 7.15 (s, 2H, H_ArOH_-2, H_ArOH_-6), 5.20 (s, 1H, OH), 4.83 (dd, *J* 8.8, 8.8, 1H, H-3), 3.86 (s, 3H, OC*H*_*3*_), 3.76 (dd, *J* 8.8, 7.8, 1H, H-3a), 3.56 (d, *J* 7.8, 1H, H-6a), 2.86 (d, *J* 8.8, 1H, N*H*), 1.69 (s, 3H, C*H*_*3*_), 1.43 (s, 18H, C (C*H*_3_)_3_). ^13^C NMR (100 MHz; CDCl_3_): **δ** 172.91, 172.13, 171.14, 153.77, 135.77 (2C), 125.72, 123.70 (4C), 107.61, 107.39 (2C), 107.16, 68.07, 63.66, 56.76, 52.79, 51.28, 34.32, 30.15 (3C), 23.91. Anal. Calcd. for C_29_H_32_F_4_N_2_O_5_: C, 61.70; H, 5.71; N, 4.96. Found: C, 61.52; H, 5.76; N, 5.18.

#### 2.1.3. N^6^-(2-((2-Hydroxyphenyl)thio)acetyl)-*L*-lysine (**3**)

Solution of 0.700 g (4.8 mmol) of *L*-lysine in 40 mL of 80% aqueous methanol was added dropwise to the stirred solution of 1.500 g (9.0 mmol) of benzo[*b*][1,4]oxathiin-2(3*H*)-one [9] in 12 mL of methanol at +40°C. The reaction mixture was stirred for 10 h at +50°C. After cooling to rt the reaction mixture was treated with 150 mL of acetone. The precipitate was filtered, rinsed with 15 mL of acetone, and dried under vacuum. Yield 40% (1.680 g), beige powder, mp 198-199°C. ^1^H NMR (400 MHz; D_2_O): **δ** 7.29 (dd, *J* 8.0, 1.5, 1H, Ar), 7.18 (td, *J* 8.0, 1.5, 1H, Ar), 6.86 (d, *J* 8.0, 1H, Ar), 6.83 (t, *J* 8.0, 1H, Ar), 3.55 (t, *J* 6.5, 1H), 3.41 (s, 2H), 2.95 (t, *J* 6.5, 1H), 1.80-1.00 (m, 8H). ^13^C NMR (100 MHz; DMSO-d_6_): **δ** 171.59, 168.79, 156.46, 130.73, 128.12, 121.97, 119.75, 115.75, 54.39, 39.19, 36.80, 31.03, 29.06, 22.83. Anal. Calcd. for C_14_H_20_N_2_O_4_S: C, 53.83; H, 6.45; N, 8.97. Found: C, 54.14; H, 6.52; N, 8.75.

#### 2.1.4. Methyl (2-((2-Hydroxyphenyl)thio)acetyl)-*L*-prolinate (**4**)

Solution of 1.600 g (12.4 mmol) of methyl *L*-prolinate in 10 mL of CCl_4_ was added dropwise to the stirred solution of 2.000 g (12.0 mmol) of benzo[*b*][1,4]oxathiin-2(3*H*)-one [9] in 35 mL of CCl_4_, and stirred for 6 h at +50°C. After cooling to rt the precipitate was filtered, rinsed with 5 mL of CCl_4_, and dried on air. Yield 95% (3.380 g), yellowish powder, mp 153-154°C, [*α*]_*D*_^20^ –51.43 (*c* 1.23, CH_2_Cl_2_). ^1^H NMR (400 MHz; CDCl_3_): **δ** 9.19 (br.s, 1H, O*H*), 7.52–7.48 (m, 1H, Ar), 7.29–7.24 (m, 1H), 7.00–6.96 (m, 1H, Ar), 6.83–6.78 (m, 1H, Ar), 4.53 (dd, *J* 8.5, 3.7, 1H), 3.73 (s, 3H, OC*H*_3_), 3.68–3.64 (m, 1H), 3.64–3.54 (m, 2H), 3.43–3.38 (m, 1H), 2.22–1.88 (m, 4H). Anal. Calcd. for C_14_H_17_NO_4_S: C, 56.93; H, 5.80; N, 4.74. Found: %: C, 57.02; H, 5.73; N, 4.59.

### 2.2. Animal Tests

The study was carried out on 42 rats weighing 200–220 g in accordance with guidelines of Animal Ethical Research Committee of Taras Shevchenko National University of Kyiv. The animals had been deprived of food for 24 hr prior to the experiments with an easy access to water. Injuries (ulcers, erosion, and hemorrhages) in gastric mucosa (GM) were caused by water-immersion restraint stress (WIRS) [10, 11]. The animals were immobilized in perforated metal tubes with transparent perforated Plexiglas windows at the ends. The tubes had been vertically immersed in a bath with water at 22–23°C for 3 hours up to the level of animal necks. The animals were divided into 7 groups (6 rats per group). In group **I** (intact control) rats were injected intraperitoneally (IP) with placebo (0.4 mL of physiological saline). In group **II** (stress-control) animals received IP 0.4 mL of physiological saline 30 min before WIRS. In group **III** animals were IP injected with 0.4 mL solution aqueous dimethyl sulfoxide (DMSO) (10 *μ*L of DMSO in 1 mL of saline) 30 min before immobilization. The rats of **IV**–**VII** groups were injected IP with 0.4 mL solutions of small-molecule agents **1**, **2**, **3**, **4**, respectively, in dose 1 mg/kg. The compounds **1** and **3** are water soluble. Compounds **2** and **4** were dissolved in 10 *μ*L DMSO and 1 mL of saline was added to each solution. The lethal dose of urethane (3 g/kg, IP) was used for causing the rats death. The stomach was removed, cut along the lesser curvature, turned out (mucosa out), and thoroughly washed with physiological saline. GM was carefully examined visually using a magnifying glass equipment. The area of ulcers and length of erosions were estimated. The character of hemorrhage was assessed on five-point scale.

### 2.3. Measurements of Lipid Peroxidation

The content of primary LPO products (diene conjugates) in GM homogenate was measured spectrophotometrically [12]. The content of thiobarbituric acid reactive substances (TBARS) was studied by the reaction with thiobarbituric acid [13]. The concentration of Schiff bases, final LPO products, was measured fluorometrically [14].

### 2.4. Measurements of Enzymes Activities

The antioxidant system state was estimated by the activities of superoxide dismutase (SOD) [15], catalase [16] and xanthine oxidase [17] in GM homogenate.

### 2.5. Data Analysis

The normality of data distribution was verified by Shapiro-Wilk *W*-test. At the normal distribution the samples were compared by Student's *t*-test. Ulcerative and erosive lesions data not corresponding to the normal distribution were compared by Mann-Whitney test. Data are presented as means ± SE (M ± m ). Significant differences were considered at *P* ≤ 0.05.

## 3. Results

### 3.1. Characterization of Compounds **1–4**

The purity of small-molecule compounds **1**–**4** was confirmed by TLC and elemental analysis data. Structural assignments were made by NMR spectra analysis and correlation of spectral characteristics with earlier established parameters [8, 9, 18, 19].

### 3.2. Gastroprotective Activity of Compounds **1–4** in Rats WIRS Ulceration

**Figure 2:**
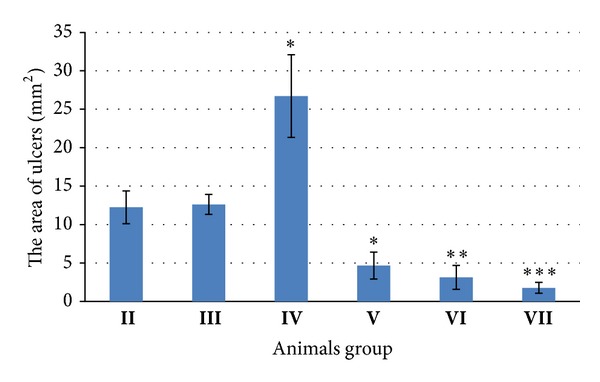
The influence of small-molecule agents **1**, **2**, **3**, and **4** (dose 1 mg/kg, intraperitoneally) on the area of gastric mucosa ulceration caused by stress in rats: **II**: stress-control group; **III**: stress-control group treated with aqueous DMSO (10 *μ*L of DMSO in 1 mL of saline); **IV**: compound **1**; **V**: compound **2** in aqueous DMSO; **VI**: compound **3**; **VII**: compound **4** in aqueous DMSO. *P < 0.05; **P < 0.01; ***P < 0.001.

The intact control (sham) group **I** rats had no stomach injuries under analysis. WIRS during 3 hours caused the development of ulcers (area 12.25 ± 2.14 mm^2^ per stomach), erosions (length 0.66 ± 0.6 mm), and hemorrhages (2.5 ± 0.32 points) in GM in the stress-control group **II** (Figure 2). Development of injuries was accompanied by intensification of LPO that was displayed by the increase of diene conjugates by 59.0% (*P* < 0.01), TBARS by 139.0% (*P* < 0.01), and Schiff bases by 59.0% (*P* < 0.01) in the stress-control group **II** against the intact control group **I** (Table 1). Analysis of enzymatic activity of the stress-control group **II** animals revealed increase of catalase activity by 86.1% (*P* < 0.01) and decrease of SOD and xanthine oxidase activity by 52.6% (*P* < 0.01) and 38.3% (*P* < 0.01), respectively, against the sham group **I** (Table 2). Pretreatment of the animals with dipeptide analog **1** increased the area of WIRS-induced ulcers by 117.0% (*P* < 0.05) and hemorrhages by 46.7% (*P* < 0.05) in comparison with the stress-control group **II**. The injection of compounds **2**, **3**, and **4** solutions to rats before application of stress led to reducing of the ulcers area by 62.0% (*P* < 0.05), 74.0% (*P* < 0.01), and 86.0% (*P* < 0.001), respectively, compared to group **II** (Figure 2). The formations of erosions and hemorrhages were not detected under the treatment of rats with the single dose of agents **2**, **3**, or **4** (corresponding animals groups **V**, **VI**, and **VII**). Amino acid derivatives of (2-hydroxyphenyl)thioacetic acid **3** and **4** were selected for the further investigations as the drugs with the most pronounced cytoprotective properties.

### 3.3. Influence of Compound ** 3** on LPO and Antioxidative Enzymes in Rats GM

Pretreatment of rats with lysine derivative **3** before WIRS provided a definite effect on the level of LPO (Table 1). The concentration of diene conjugates was reduced in group **VI** by 14.4% (*P* < 0.05), the amount of TBA-active products decreased by 45.8% (*P* < 0.01), and the amount of Schiff bases decreased by 22.9% (*P* < 0.05) compared to the stress-control group **II**. The content of secondary and tertiary LPO products in rats GM after pretreatment with compound **3** is at the same levels as in the intact control group **I** (Table 1). Xanthine oxidase and catalase activities under the influence of compound **3** were also restored to the levels of the intact control group (Table 2). At the same time SOD activity in the animal group **VI** lowered by 31.6% (*P* < 0.01) in contrast with the intact control group **I** but increased against the stress-control group **II** by 44.4% (*P* < 0.05).

### 3.4. Influence of Compound ** 4** on LPO and Antioxidative Enzymes in Rats GM

Injection of proline derivative **4** prior WIRS decreased the formation of diene conjugates, TBARS, and Schiff bases by 32.0% (*P* < 0.01), 41.6% (*P* < 0.01), and 7.0% (*P* > 0.05), respectively, against the stress-control group **II** (Table 1). At the same time contents of diene conjugates and TBARS after pretreatment with compound **4** approach the corresponding levels for the sham group **I**. The concentration of Schiff bases after pretreatment with compound **4** is not normalized and was higher by 47.6% (*P* < 0.05) as compared with the intact control group **I**. Pretreatment of animals with compound **4** reduced catalase activity by 21.7% (*P* < 0.05) against the enzyme activity level in group **II** (Table 2). At the same time catalase activity was still higher by 45.8% (*P* < 0.05) than in the intact control group **I**. Xanthine oxidase activity under influence of compound **4** was restored to the level of the intact control group **I**. The activity of SOD was affected poorly by compound **4** in contrast with the control groups **II** and **III**.

## 4. Discussion

Exposition to physical and psychological stresses triggers diverse pathological disorders in humans, including gastrointestinal diseases. Hydrogen ions and oxygen radicals are the main inducers of harmful GM lesions at the molecular level. Ischemic conditions in GM arise due to vascular compression caused by stress that leads to increased ROS generation [4]. Especially highly damaging HO^•^ depletes cellular thiol antioxidants and oxidizes biomacromolecules inducing cell death. Some antioxidants and HO^•^ scavengers prevented stress-induced ulceration indicating a possibility of these agents application for SRMD cure [4]. Small-molecule drugs, rebamipide [5], melatonin [6], and omeprazole [7], also exert antioxidant properties during gastric ulcer treatment. Studied compounds **1**–**4** were selected from the MSU in-house compound library inspiring by structural fragments with known antioxidative activity according to the literature data (Figure 1). Namely, dipeptide derivative **1** is a structural analog of Pro-Gly-Pro tripeptide possessed with gastroprotective properties [20, 21]. Compounds **2**–**4** contain sterically hindered [8] or intramolecular hydrogen bond modified phenolic fragments [22] occurring in the majority of natural and artificial antioxidants. In distinction from the referred analogs small molecules **1**–**4** are characterized with well-defined stereochemistry and ample opportunities of structural and physicochemical properties modification for potential subsequent activity variation and improvement. The dose 1 mg/kg for the studied compounds **1**–**4** was adopted as the reference point of known gastroprotective agents [5–7, 20, 21].

Since compounds **2** and **4** required DMSO for solubilization, an additional stress-control group **III** was introduced in our study. The animals of this group received 1% aqueous DMSO before WIRS. DMSO protective effect on GM ulceration was reported under long-term prophylactic application [23]. In our study pretreatment of animals with low-dose DMSO did not induce substantial deviations of ulceration and biochemical data against the saline control group **II** (Figure 2, Tables 1 and 2).

From four studied small-molecule agents, three compounds revealed pronounced reduction of gastric ulcers, erosion, and hemorrhages under stress-related conditions and preventive single-dose application. Bicyclic pyrrolidine **2** reduced stomach ulceration area from 12.62 mm^2^ to 4.66 mm^2^ (2.7 times decreasing), lysine derivative **3** reduced stomach ulceration area from 12.25 mm^2^ to 3.13 mm^2^ (3.9 times decreasing), and proline derivative **4** reduced stomach ulceration area from 12.62 mm^2^ to 1.77 mm^2^ (7.1 times decreasing). All three active compounds prevented formation of stress-induced erosions and hemorrhages in stomach. It is worth to note that all three active compounds include phenolic fragment in molecular structure.

Intensification of oxidative processes in rats stomach under applied stress conditions are unequivocally confirmed by increasing of all LPO parameters in stress-control groups **II** and **III** (Table 1). Amount of conjugated dienes, TBARS, and Schiff bases in rats GM significantly increased under stress conditions up to 59%–139% compared with the sham group **I**. When the stress-exposed animals were pretreated with thioacetamide derivatives **3** and **4**, decreasing of all LPO components in GM homogenates was observed indicating the cytoprotective action of the agents (Table 1, animals groups **VI** and **VII**).

Proteins of antioxidative system were examined in all examined conditions to elucidate potential mechanism of gastroprotective compounds **3** and **4** action. Activity of SOD, effective superoxide scavenger, decreased in the stress-control groups **II** and **III** by 52.6% (*P* < 0.01) and 47.4% (*P* < 0.01), respectively, compared to the intact control group **I** (Table 2). Invertedly catalase activity in both stress-control groups **II** and **III** increased by 86.1% (*P* < 0.01) and 62.7% (*P* < 0.01), respectively, in comparison with the intact control group **I** (Table 2). Seemingly these data indicate that effective concentration of O_2_^•−^ is higher due to the lack of SOD activity and H_2_O_2_ is generated in increased amount that requires additional catalase in the stress-subjected organisms. Both factors should increase oxidative tissue damage and subsequent ulceration. Activity of xanthine oxidase capable of reducing molecular oxygen to both O_2_^•−^ and H_2_O_2_ lowered in both stress-control groups **II** and **III** by 38.3% (*P* < 0.01) and 26.4% (*P* < 0.01) against the sham group **I** (Table 2). Observed descension of xanthine oxidase activity correlates with ischemic conditions presented in the literature [24] and could partially account for SOD diminishing.

Before treatment of animals, the single dose of (2-hydroxyphenyl)thioacetamide derivatives **3** and **4** (corresponding animals groups **VI** and **VII**) restored to a certain degree levels of all considered enzymes. SOD activity increased in the animals group **VI** by 44.4% (*P* < 0.05) compared to the stress-control group **II** (Table 2). SOD increasing in the animals group **VII** against the stress-control group **II** was insignificant. Catalase activity in both animals groups **VI** and **VII** is reduced by 27.3% (*P* < 0.05) and 21.7% (*P* < 0.05), respectively, compared to the stress-control group **II** (Table 2). Xanthine oxidase activity in both animals groups **VI** and **VII** enhanced by 52.3% (*P* < 0.05) and 46.2% (*P* < 0.05), respectively, against the stress-control group **II**.

## 5. Conclusion

Methods of gastrointestinal diseases healing may depend on etiology of damage events. Drugs modulating organism oxidative status through interaction with particular biological targets provide valuable alternatives or additions to the existing therapeutics. Marked gastroprotective activity of (2-hydroxyphenyl)thioacetamides **3** and **4** and preliminary characterization of their influence on antioxidant system enzymes provide basis for the development of novel chemotypes of antiulcer agents.

## Figures and Tables

**Table 1:** Influence of compounds **3** (group **VI**) and **4** (group **VII**) (1 mg/kg, IP, 30 min before WIRS) on the content of lipid peroxidation products in homogenates of rats gastric mucosa (*n* = 6, M ± m).

Animals group	**I**	**II**	**III**	**VI**	**VII**
Conjugated dienes (nmol mg protein^−1^)	326.11 ± 25.78	518.33 ± 26.72**	465.56 ± 14.67**	443.89 ± 15.44^∗∗#^	352.22 ± 27.17^##^
TBA-reactive substances (nmol mg protein^−1^)	69.4 ± 9.24	165.70 ± 12.25**	130.10 ± 10.26**	89.73 ± 6.55^##^	98.26 ± 8.87^##^
Schiff bases (units mg protein^−1^)	61.45 ± 4.68	97.53 ± 6.38**	92.17 ± 7.20**	75.17 ± 3.24^#^	90.67 ± 4.52*

*P < 0.05, **P < 0.01 compared to the intact control group **I**; ^#^ *P* < 0.05, ^##^ *P* < 0.01 compared to the stress control group **II**.

**Table 2:** Influence of compounds **3** (group **VI**) and **4** (group **VII**) (1 mg/kg, IP, 30 min before WIRS) on the activity of antioxidant enzymes in homogenates of rats gastric mucosa (*n* = 6, M ± m).

Animals group	**I**	**II**	**III**	**VI**	**VII**
SOD (units min^−1^ mg protein^−1^)	0.19 ± 0.01	0.09 ± 0.01**	0.10 ± 0.01**	0.13 ± 0.01^∗∗#^	0.12 ± 0.01**
Catalase (nmol min^−1^ mg protein^−1^)	5.83 ± 0.66	10.85 ± 0.79**	9.48 ± 0.56**	7.89 ± 0.46^#^	8.50 ± 0.70^∗#^
Xanthine oxidase (nmol min^−1^ mg protein^−1^)	70.00 ± 5.20	43.20 ± 2.60**	51.50 ± 2.50**	65.80 ± 2.10^##^	63.20 ± 2.40^##^

*P < 0.05, **P < 0.01 compared to the intact control group **I**; ^#^ *P* < 0.05, ^##^ *P* < 0.01 compared to the stress control group **II**.
